# Integrating a Comprehensive Cancer Genome Profiling into Clinical Practice: A Blueprint in an Italian Referral Center

**DOI:** 10.3390/jpm12101746

**Published:** 2022-10-20

**Authors:** Camilla Nero, Simona Duranti, Flavia Giacomini, Angelo Minucci, Luciano Giacò, Alessia Piermattei, Maurizio Genuardi, Tina Pasciuto, Andrea Urbani, Gennaro Daniele, Domenica Lorusso, Raffaele Pignataro, Giampaolo Tortora, Nicola Normanno, Giovanni Scambia

**Affiliations:** 1 Dipartimento per le Scienze Della Salute Della Donna, del Bambino e di Sanità Pubblica, UOC Ginecologia Oncologica, Fondazione Policlinico Universitario Agostino Gemelli IRCCS, 00168 Roma, Italy; 2 Faculty of Medicine and Surgery, Università Cattolica del Sacro Cuore, 00168 Roma, Italy; 3 Direzione Scientifica, Fondazione Policlinico Universitario Agostino Gemelli IRCCS, 00168 Roma, Italy; 4 Genomics Core Facility, Gemelli Science and Technology Park (G-STeP), Fondazione Policlinico Universitario Agostino Gemelli IRCCS, 00168 Roma, Italy; 5 Bioinformatics Core Facility, Gemelli Science and Technology Park (G-STeP), Fondazione Policlinico Universitario Agostino Gemelli IRCCS, 00168 Roma, Italy; 6 Dipartimento per le Scienze Della Salute Della Donna, del Bambino e di Sanità Pubblica, UOC Anatomia Patologica Generale, Fondazione Policlinico Universitario Agostino Gemelli IRCCS, 00168 Roma, Italy; 7 Dipartimento per le Scienze di Laboratorio e Infettivologiche, UOC Genetica Medica, Fondazione Policlinico Universitario Agostino Gemelli IRCCS, 00168 Roma, Italy; 8 Data Collection Core Facility, Gemelli Science and Technology Park (G-STeP), Fondazione Policlinico Universitario Agostino Gemelli IRCCS, 00168 Roma, Italy; 9 Dipartimento per le Scienze di Laboratorio e Infettivologiche, UOC Chimica, Biochimica e Biologia Molecolare Clinica, Fondazione Policlinico Universitario Agostino Gemelli IRCCS, 00168 Roma, Italy; 10 UOC Fase 1, Direzione Scientifica, Fondazione Policlinico Universitario Agostino Gemelli IRCCS, 00168 Roma, Italy; 11 Direzione Sanitaria, Fondazione Policlinico Universitario Agostino Gemelli IRCCS, 00168 Roma, Italy; 12 Dipartimento di Scienze Mediche e Chirurgiche, UOC Oncologica Medica, Fondazione Policlinico Universitario Agostino Gemelli IRCCS, 00168 Roma, Italy; 13 Cell Biology and Biotherapy Unit, Istituto Nazionale Tumori “Fondazione Giovanni Pascale”, IRCCS, 80131 Napoli, Italy

**Keywords:** genomics, cancer, target therapy, comprehensive cancer genome profiling, bioinformatics

## Abstract

The implementation of cancer molecular characterization in clinical practice has improved prognostic re-definition, extending the eligibility to a continuously increasing number of targeted treatments. Broad molecular profiling technologies better than organ-based approaches are believed to serve such dynamic purposes. We here present the workflow our institution adopted to run a comprehensive cancer genome profiling in clinical practice. This article describes the workflow designed to make a comprehensive cancer genome profiling program feasible and sustainable in a large-volume referral hospital.

## 1. Introduction

The introduction of molecular tumor profiling in the management of cancer patients is progressively forcing a reappraisal of the approach to cancer diagnosis and care.

The possibility of prognostic re-definition and the availability of an increasing number of biomarker-driven targeted therapies have made the implementation of molecular characterization into clinical practice an essential need. Most of the advances in precision oncology rely on genomic sequencing and in particular on next-generation sequencing (NGS) approaches.

The objective of personalized/precision medicine is the application of genomic information in order to define appropriate interventions (screening, prevention and treatment) that could benefit both patients and health authorities in terms of clinical and healthcare outcomes [1].

In 2020, the European Society of Medical Oncology (ESMO) recommended the implementation of comprehensive cancer genome profiling (CGP) for selected tumor types at least in academic centers [2]. However, integrating CGP in the clinical workflow can be challenging in terms of infrastructure requirements, methodologies, timing, resources, expertise, multidisciplinary interactions and reimbursement policies [3].

Moreover, discrepancies among different NGS assays, the lack of standardized operative procedures and the heterogeneity among clinical frameworks are major issues to be addressed in order to assess the real value of genomic testing for precision medicine.

Aware of all these issues, the Fondazione Policlinico Universitario Agostino Gemelli IRCCS (FPG), a referral Italian research hospital, launched a CGP program (ID: FPG500, ethical committee approval number 3837) including 10 different cancer types. The program extends genomic assessment to more than 500 genes in a streamlined in-house process at no extra cost to the public healthcare system.

Measurable outcomes include the feasibility of CGP from formalin-fixed paraffin-embedded (FFPE) and cytological specimens, turnaround time (TAT), time to therapy initiation, access to available target treatment (indication and off-label), enrollment in clinical trials and the rate of indications for referral to genetic counseling.

**Figure 1:**
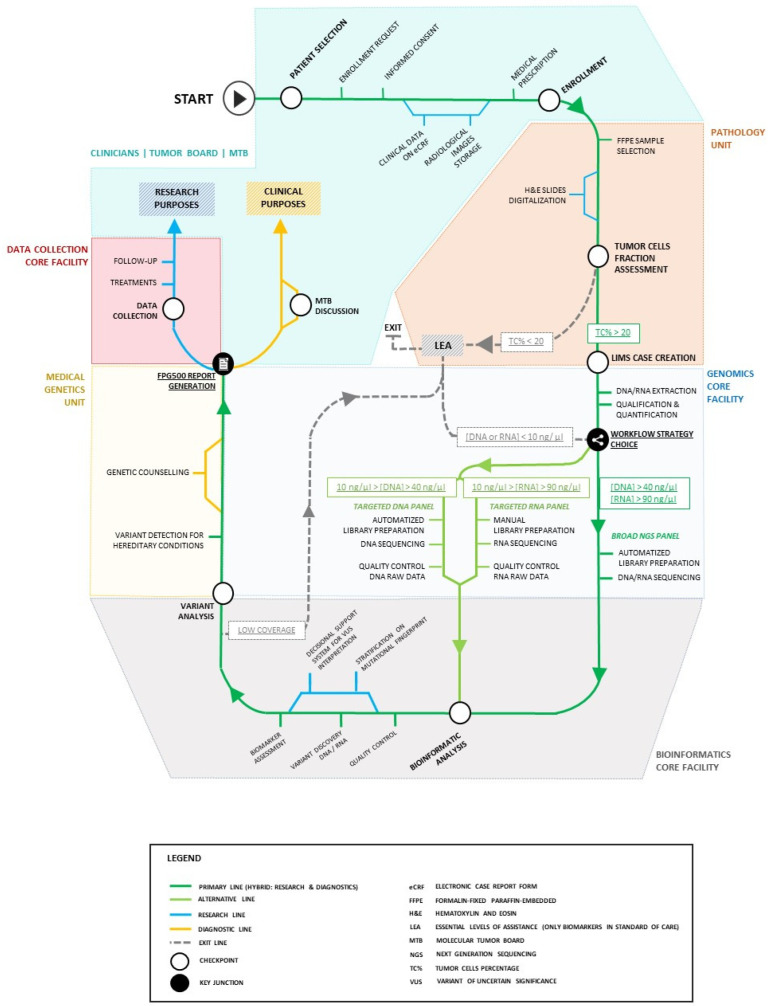
CGP journey.

The hybrid nature (diagnostic and research) of FPG500 might represent a blueprint for healthcare optimization in which a clinical process is turned into an opportunity for research to improve the care of cancer patients in the future. Patient, clinician and researcher journeys are shown in Figure 1.

## 2. Inclusion Criteria

Patients can be included if affected by malignancies for which molecular characterization should be warranted according to national and international guidelines in specific clinical settings (lung, ovarian, prostate, pancreas, melanoma, breast, gastrointestinal stromal tumor (GIST), colorectal, thyroid, endometrial cancers) and for which reimbursement policies are already in place in Italy (see Table 1).

Patients are selected during the multidisciplinary tumor board (MDT) sessions but are also referred by clinicians outside FPG. A molecular care manager has been selected to attend MDT meetings and together with referral oncologists for each cancer type is in charge of connecting patients, clinicians and study coordinators, smoothing the whole process. Patients are informed that the primary aim of CGP is to assess the presence of targetable mutations for which target therapies are already available as well as additional genomic alterations which might be relevant for research purposes. Moreover, variants associated with the risk of hereditary tumors can be found and will need subsequent confirmation of germline origin. All documentation (signed informed consent, properly completed application form, medical history and medical prescription for genomic profiling) is provided by email and revised by the program coordinator before enrolment. Updates regarding patient enrolment are provided every week by email to clinicians, study coordinators and care managers to ensure continuous communication. Finally, weekly meetings are held among the whole team to discuss major and minor issues to be addressed.

## 3. Infrastructure

The whole analytical process (wet and dry steps) is managed through an all-in-one digital platform. The SLIMS (Agilent), designed for NGS, combines a laboratory information management system (LIMS) and an electronic laboratory notebook (ELN) to ensure data tracking and managing. In this workflow, all samples, from DNA/RNA extraction to sequencing, are tracked in an intuitive interface, and all laboratory protocols are integrated in order to follow each step of sample processing. The SLIMS is also integrated with instruments such as the Microlab STAR-Hamilton, for automated library preparation, and the Illumina Novaseq6000 for the sequencing. The platform has been further customized to be fully integrated with the hospital information system (SIO). In detail, input and output data between the SLIMS and the SIO are exchanged through Health Level Seven (HL7) messages, the most widely used messaging standard for the exchange of patient care and clinical information.

## 4. Sample Preparation and Sequencing

Samples from surgeries, core needle biopsies, fine needle aspiration or cytology are reviewed by dedicated pathologists in order to assess tumor cell (TC) fraction. The minimum requirement to access nucleic acid extraction is a TC of at least 20%, with an optimal value being >30%.

Once samples have been selected, Systematized Nomenclature of Medicine (SNOMED) codes for diagnosis and specimens are assigned on the dedicated platform. All H&E slices undergo digitalization before nucleic acid extraction.

A semi-automated process takes place for simultaneous DNA/RNA extraction (Qiacube Connect, Qiagen), DNA fragmentation (Covaris M220, Euroclone, Woburn, MA, USA), DNA/RNA quantification/qualification (Infinium kit, Illumina, San Diego, CA, USA and TapeStation 2200, Agilent, Santa Clara, CA, USA), automated library preparation (Microlab STAR-Hamilton, Reno, NV, USA) and sequencing (Novaseq6000-Illumina).

Profiling is performed with the TruSight Oncology 500 high throughput (TSO500HT, Illumina), an assay that analyzes both DNA and RNA, identifying single nucleotide variants (SNVs), insertions/deletions (indels) and copy number variations (CNVs) in 523 genes as well as known and unknown fusions and splicing variants in 55 genes. In addition, it provides genomic “signatures” such as microsatellite instability (MSI) and tumor mutational burden (TMB), which is a measure of the total number of somatic mutations present in the sequenced genome. A validation process of the workflow was successfully run before implementing the test in the program.

Samples not reaching the required quantity threshold for TSO500HT (DNA or RNA ≥ 40 ng) and for which no other specimens are available undergo Oncomine Focus Assay (OFA) (Thermo Fisher, Waltham, MA, USA) and Archer’s FusionPlex Lung Panel (AFL) (Archer, Boulder, CO, USA) for DNA and RNA evaluation, respectively.

Samples not reaching 20 ng of DNA/RNA are discussed with referral clinicians to evaluate either a re-biopsy or testing with standard-of-care techniques limited to the biomarkers approved for clinical practice and included in the Essential Levels of Assistance (LEA). Liquid biopsy is considered when no other option is available.

For TSO500HT, raw sequencing data are processed by the Illumina Software TSO500 v2.2 Local App and then, through a customized analysis pipeline (https://github.com/lucianogiaco/lianne accessed on 09 August 2022), are sent to the Clinical Genomics Workspace software platform by Pierian Dx for variant interpretation and reporting.

Samples are sequenced with a mean depth of > 500×. The minimum coverage accepted for variant calling is 100× on 90% of sequenced targeted regions and at least 250× on hotspot regions. Samples that do not meet these criteria are re-sequenced or re-extracted from the biological specimen.

After sequencing, an accurate quality control (QC) at nucleotide resolution is performed using a custom tool integrated in the bioinformatic pipeline (https://github.com/fernandoPalluzzi/VarHound accessed on 09 August 2022). Afterward, the report is generated and data are filtered for non-synonymous, exonic variants and splice site variants in the flanking regions showing an allele frequency > 5%. Variants with a population minor allele frequency of more than 1% in 1000 Genomes and dbSNP are excluded since they are considered known polymorphisms.

Genomic alterations are reported according to the Human Genome Variation Society (HGVS) nomenclature [63] and classified according to the Association for Medical Pathology (AMP), American Society of Clinical Oncology and College of American Pathologists classification system into tiers IA, IB, IIC, IID, III and IV [64]. These tiers are stratified according to clinical usefulness (“actionability” for clinical decision-making regarding diagnosis, prognosis, treatment options and carrier status) and data previously reported in the scientific literature.

For samples not fulfilling the requirements for TSO500 sequencing, targeted panels requiring a lower DNA/RNA input are used. The Oncomine Focus Assay (OFA) is a targeted NGS assay that enables the simultaneous detection of multiple variants across 35 tumor-related genes from DNA using amplicon-based enrichment. Library preparation, amplification and ligation steps are performed in line with the OFA protocol. Sequencing is performed using semiconductor sequencing technology (Ion S5, Thermo Fisher Scientific). Data analysis is performed using the Torrent Server Variant Caller and the Ion Reporter Software (Thermo Fisher Scientific, Darmstadt, Germany).

The Archer Fusion Lung (AFL) NGS assay is designed to detect key fusions in 17 genes, skipping events in EGFR vIII and MET exon 14, and select point mutations in 14 key lung cancer-associated genes. The AFL uses Archer’s Anchor Multiplex PCR chemistry to target regions of interest. Because of the use of one gene-specific primer and one universal primer, both known and unknown gene fusion partners can be detected. Reagent preparation and DNA synthesis, ligation and amplification are performed according to the official assay protocol.

Libraries are multiplexed for sequencing on an Illumina MiSeq. Data are analyzed with the Archer Analysis software.

The genomic report is reviewed by molecular biologists, bioinformaticians, pathologists and geneticists. When somatic variants have a possible germline correlation, genetic counseling is indicated. Specifically, patients will be referred to genetic assessment when a pathogenic or likely pathogenic variant with variant allele frequency (VAF) > 20% is identified in a clinically actionable cancer predisposition gene. Variants in genes known to be common targets of somatic hits, such as TP53 in all tumors or PTEN in endometrial carcinoma, will not be considered for genetic counseling unless suggested by clinical and/or molecular characteristics and family history [65,66].

An institutional Molecular Tumor Board (MTB) is available to help clinicians interpret and manage complex genomic profiling reports. In particular, the MTB discusses all cases with documented variants for which no approved drugs exist to verify the availability of clinical trials or off-label use of drugs (i.e., expanded access program, compassionate use, etc.). The MTB is scheduled every 2 weeks and involves not only a core team (oncologist, pathologist, molecular biologist, geneticist, methodology expert, bioinformatician, hospital pharmacist, radiation oncologist, phase I trial oncologist, clinical epidemiologist, psychologist) but also optional professionals (surgeon, internal/external treating physician, etc.) included for case-specific discussions. The attending physician presents the case, and the board expresses a recommendation that is uploaded into the patient’s electronic chart.

## 5. Data Collection

Clinical, family history, radiological, pathological, therapeutic and follow-up data for each enrolled patient are collected in a dedicated electronic case report form (eCRF) [67,68]. Access to the system is restricted to the study personnel by username and password with a two-step login authentication. Moreover, digitalized hematoxylin and eosin (H&E) slides and radiological images are systematically stored for each patient. Data processing takes place in compliance with current Italian and European legislation regarding General Data Protection Regulation. The CRF is implemented according to validation, branching and skipping logic criteria. The accuracy, completeness, consistency and integrity of data collection are addressed and/or monitored through several instruments and functions, and data quality rules are executed to check for discrepancies in study data. Different user privileges are given to users according to the data minimization principle.

## 6. Strengths and Limitations

Major challenges to the implementation of genomic profiling into routine care have been outlined and only partially addressed in this program; they are detailed as follows:

1. The majority of available CGP solutions are not In Vitro Diagnostics (IVD)-marked by Conformité Européenne (CE); this mark not only guarantees standards for quality and efficacy but is also required for reimbursement of diagnostic tests by many European health authorities. Academic centers can use non-IVD solutions if internally validated, benefitting also from the use of high-performance benchtop sequencing platforms which are not yet IVD-marked [69]. As a matter of fact, without the simultaneous certification of those platforms, the adoption of CE-IVD kits would not be efficient for centers with high volumes of patients. In the near future, we expect companies themselves to develop assays as well as instruments IVD-marked as requested by EU regulation. The transition should be supported by both institutions and companies to make it affordable and smooth.
2. The complexity and the high costs of adopting an in-house advanced genomic platform and dedicated team for data interpretation make the spread of genomics laboratories in healthcare institutions quite unlikely, even in privileged countries. In this scenario, a smooth, fast and safe transfer of patient samples and data should be planned across institutions and regions. A centralization of advanced genomics diagnostics in a sort of hub and spoke model would also allow efficient monitoring from health authorities encouraging accreditation processes in compliance with the EU General Data Protection Regulation (GDPR).
3. High-quality clinical genomic data registries should be pursued by health authorities in order to evaluate the real impact of CGP in oncology. Cross-test comparison and validation tests to confirm mutations should be encouraged to evaluate the most cost-effective and efficient solutions available.
4. Alternative strategies in case of low-quality or low-quantity material should be implemented. Referral centers should conduct studies dedicated to reinforcing evidence on the feasibility and reliability of liquid biopsy, which is more feasible in clinical practice, and to monitoring the evolution of the disease at several time points.
5. Therapeutic and clinical implications of CGP for patients remain critical issues. Dedicated resources and favorable policies to fast-track research advances into clinical practice are required. Moreover, software dedicated to variant calling and clinical trial matching should be improved and customized based on local needs and national/international regulation authorities’ policies.
6. Educational programs in genomics for healthcare professionals and physicians involved in cancer care should be integrated into training curricula.
7. The availability of an MTB is fundamental for transversal education and for sharing therapeutic decisions in a multidisciplinary context.
8. The identification of variants potentially associated with hereditary conditions is a plus for cancer prevention, but successful management of incidental findings including access to genetic counseling takes time and requires additional in-person consultations for dedicated blood sampling. It could be faster and more effective to confirm the germline origin of variants immediately after their identification by taking a blood sample at the time of enrollment. A study amendment on this topic has recently been considered and will soon be discussed.
9. Economic resources should be dedicated to the automation of the large-scale sequencing process. This could not only reduce variability but also significantly reduce timing. CGP in fact allows almost the whole picture of genomic alterations to be obtained at once but takes much longer than targeted assays. Integrating CGP within patients’ diagnostic workup could be challenging for an optimal time to therapy initiation. Patient clinical conditions should always guide physicians to the most appropriate diagnostic option (CGP vs. small panels).
10. Strategies for genomics data storage should be improved to avoid a bottleneck in the implementations of testing.
11. Availability of and accessibility to targeted agents is a mainstay of the global process and the principal output from the clinical patient perspective.

## 7. Conclusions

It seems clear that a sustainable and effective implementation of CPG within the diagnostic workup of cancer patients is challenging and requires improved policies and processes to concretely maximize benefits for present and future oncological patients and the healthcare system.

## Figures and Tables

**Table 1:** Included malignancies and related targets.

Tumor Type	Target	Setting	References
Breast	PIK3CA	Hormone receptor (HR)-positive, human epidermal growth factor receptor 2 (HER2)-negative, locally advanced or metastatic breast cancer after disease progression following endocrine therapy as monotherapy	[4]
Lung	EGFR	Metastatic	[5,6,7,8,9,10,11,12,13,14]
	ALK	Metastatic	[15,16,17,18,19]
	ROS1	Metastatic	[20,21,22]
	BRAF	Metastatic	[23,24,25]
	NTRK	Metastatic	[26,27,28]
	RET	Metastatic	[29]
Ovary	BRCA 1/2	All patients with non-mucinous and non-borderline ovarian, fallopian tube or primary peritoneal cancer	[30]
Pancreas	BRCA 1/2	Metastatic	[31,32,33]
	NTRK	Metastatic	[28,34]
Prostate	BRCA 1/2	Metastatic castration-resistant	[35]
Melanoma	BRAF	Metastatic or not resectable	[36,37,38,39,40,41,42,43,44]
		Resected stage III	[44]
GIST	c-kit	Locally advanced or metastatic	[45,46,47,48,49]
		Resected (Adjuvant)	[50,51,52,53,54,55]
	PDGFRα	Locally advanced or metastatic	[45,46,47,48,49]
Colorectal	KRAS	Metastatic	[56,57,58]
	NRAS	Metastatic	[56,57,58]
	BRAF	Metastatic	[59]
	NTRK	Metastatic	[28,60]
Thyroid	RET	Advanced medullary thyroid cancer	[61]
		Advanced non-medullary thyroid cancer	[61]
Endometrium	POLE	Stage FIGO I-II, any histotype	[62]
Other		Other cases in which the oncologist considers genomic profiling appropriate	

## Data Availability

Not applicable.
